# Nifuroxazide induces apoptosis and impairs pulmonary metastasis in breast cancer model

**DOI:** 10.1038/cddis.2015.63

**Published:** 2015-03-26

**Authors:** F Yang, M Hu, Q Lei, Y Xia, Y Zhu, X Song, Y Li, H Jie, C Liu, Y Xiong, Z Zuo, A Zeng, Y Li, L Yu, G Shen, D Wang, Y Xie, T Ye, Y Wei

**Affiliations:** 1 State Key Laboratory of Biotherapy/Collaborative Innovation Center for Biotherapy, West China Hospital, West China Medical School, Sichuan University, Chengdu, China; 2 Department of Pharmacy, Xinqiao Hospital, Third Military Medical University, Chongqing, China; 3 School of Medicine, Tsinghua University, Beijing, China

## Abstract

Breast carcinoma is the most common female cancer with considerable metastatic potential. Signal transducers and activators of the transcription 3 (Stat3) signaling pathway is constitutively activated in many cancers including breast cancer and has been validated as a novel potential anticancer target. Here, we reported our finding with nifuroxazide, an antidiarrheal agent identified as a potent inhibitor of Stat3. The potency of nifuroxazide on breast cancer was assessed *in vitro* and *in vivo*. In this investigation, we found that nifuroxazide decreased the viability of three breast cancer cell lines and induced apoptosis of cancer cells in a dose-dependent manner. In addition, western blot analysis demonstrated that the occurrence of its apoptosis was associated with activation of cleaved caspases-3 and Bax, downregulation of Bcl-2. Moreover, nifuroxazide markedly blocked cancer cell migration and invasion, and the reduction of phosphorylated-Stat3^Tyr705^, matrix metalloproteinase (MMP) MMP-2 and MMP-9 expression were also observed. Furthermore, in our animal experiments, intraperitoneal administration of 50 mg/kg/day nifuroxazide suppressed 4T1 tumor growth and blocked formation of pulmonary metastases without detectable toxicity. Meanwhile, histological and immunohistochemical analyses revealed a decrease in Ki-67-positive cells, MMP-9-positive cells and an increase in cleaved caspase-3-positive cells upon nifuroxazide. Notably, nifuroxazide reduced the number of myeloid-derived suppressor cell in the lung. Our data indicated that nifuroxazide may potentially be a therapeutic agent for growth and metastasis of breast cancer.

Breast cancer is the most common type of cancer among women, and its incidence is increasing worldwide. According to statistics, about 62 570 cases of breast cancer *in situ* are expected to be newly diagnosed in the United States in 2014.^1^ Moreover, the incidence of breast cancer – like most cancers – is on the rise in developing countries such as Brazil and China, as populations increasingly adopt western lifestyles.^2^ Therefore, breast cancer ranks the second most common cause of cancer death among women worldwide, with about 1.4 million new cases annually.^1, 3, 4^ Despite significant improvement in survival rates of patients with breast cancer, the disease remains a huge threat to women's health, and particularly patients with ‘triple-negative' breast cancer (TNBC), referring to cancers that express neither the estrogen receptor or progesterone receptor nor display amplification of human epidermal growth factor receptor 2, are insensitive to hormonal therapy or HER2-targeted drugs.^5, 6, 7^ Advanced TNBC confer an aggressive clinical course with a poor prognosis compared with non-TNBC.^8^ Furthermore, breast cancer is highly malignant with considerable metastatic potential, and metastatic breast cancer is a principle cause of female mortality.^9^ Unfortunately, there is currently no effective therapy to control the recurrence and metastasis of breast cancer, and therefore the development of new therapies is essential.

Signal transducer and activator of transcription 3 (Stat3) has important roles in cancer and other disease, and presents tremendous therapeutic potential.^10^ Stat3 is a point of convergence for multiple oncogenic signaling pathways. Meanwhile, Stat3 as a proto-oncogene could mediate cellular and biological processes.^10^ In a variety of human cancers, constitutively active Stat3 signaling promotes tumorigenesis and tumor progression by dysregulating the expression of key genes that control cell apoptosis (such as Bcl-2, Bcl-xl and Mcl-1), proliferation (cyclin d1, c-Myc), angiogenesis (vascular endothelial growth factor), migration, invasion or metastasis (matrix metalloproteinase 1 (MMP1), MMP7 and MMP-9).^11, 12, 13, 14^ Moreover, Stat3 is a key negative regulator of tumor immune surveillance and is critically involved in tumor accumulation of myeloid-derived suppressor cells (MDSCs), which has an important role in suppressing antitumor immune responses (S100A9).^15, 16, 17^ In breast cancer, existing evidences demonstrate that Stat3 acts as a proto-oncogene and may be associated with chemotherapeutic resistance.^12, 18^ In addition, Stat3 is constitutively activated in ~70% of breast tumors, particularly is most often associated with triple-negative tumors.^12, 14, 19^ Furthermore, orally bioavailable small-molecule inhibitor of Stat3 can inhibit tumor growth,^20^ therefore, targeting Stat3 may be an important therapeutic approach in breast cancers. Although much effort has gone into the development of Stat3 inhibitors and a number of inhibitors targeting Stat3 have been reported, so far no potent Stat3 inhibitor appears to be ready for clinical development.^21, 22, 23^

The rapid development of new safer and more effective anticancer drugs is a common goal shared by scientists and clinicians.^24^ However, drug development, from the initial lead compound to the final medication, is an expensive, lengthy and incremental process.^25^ Finding new use(s) for existing drugs is more economical and much faster than inventing a new drug, as existing drugs have safety profiles and known pharmacokinetics and have often been approved by regulatory for human use; therefore, any newly identified drugs can be rapidly evaluated in phase II clinical trials.^26^ Nifuroxazide is not currently approved for use in the USA but is used elsewhere as an antidiarrheal agent.^14^ Moreover, nifuroxazide has recently been reported as a potent inhibitor of Stat3 signaling pathway against cancer cells, though it has little effect on cells lacking Stat3 activation.^27^ However, the function of nifuroxazide on breast cancers, tumor metastasis and its related molecular mechanism have not yet been investigated.

In the current study, we observed that nifuroxazide could inhibit proliferation, induce apoptosis and suppress cell migration and invasion in breast cancer cells. Moreover, it can also repress breast tumor growth and impair formation of pulmonary metastases *in vivo* by inhibiting proliferation, inducing apoptosis, suppressing metastasis and reducing immunosuppressive cells. In conclusion, our data showed that nifuroxazide may be a potential candidate for treating breast cancer.

## Results

### Nifuroxazide inhibits breast cancer cells proliferation

**Figure 1:**
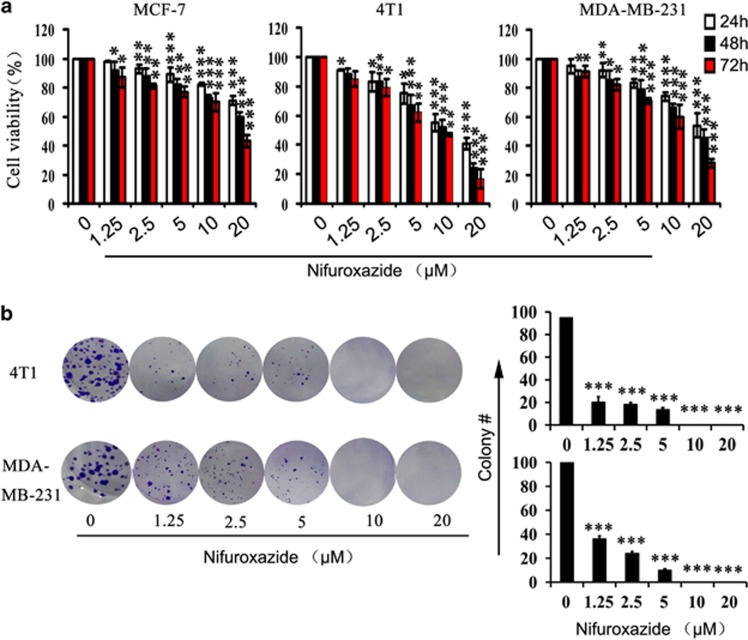
The effects of nifuroxazide on breast cancer cells viability. (**a**) Proliferation of MCF-7, 4T1 and MDA-MB-231 cells treated with various concentrations (0–20 *μ*M) of nifuroxazide for 24, 48 and 72 h, respectively. Cell viability was evaluated by MTT assay. Values represent mean±S.D., (*n*=3, in triplicate, *P<0.05; **P<0.01; ***P<0.001). (**b**) The effects of nifuroxazide (0–20 *μ*M) on colony formation in 4T1 and MDA-MB-231 cell lines for 12 days, the statistic results of colony formation assays presented as surviving colonies. Data are expressed as mean±S.D. from three experiments (*P<0.05; **P<0.01; ***P<0.001)

Because Stat3 is constitutively activated in ~70% of breast tumors, we determined the level of phospho-Stat3 (Tyr705) in three breast cancer cell lines by western blot analysis. As shown in Supplementary Figure S1a, all cancer cells had constitutively activated Stat3 as assessed by its phosphorylation status at Tyr705, especially, MDA-MB-231 and 4T1 cells. To further determine whether nifuroxazide has direct effects on breast cancer cells, we tested the assay for cell viability caused by nifuroxazide treatment on three breast cancer cell lines by 3-(4, 5)-dimethylthiahiazo(-z-y1)-3,5-di-phenytetrazoliumromide (MTT). Treatment of 4T1, MCF-7 and MDA-MB-231 cells with various concentration of nifuroxazide for 24, 48 and 72 h, respectively, resulted in a decrease in the cell viability (Figure 1a). These data suggested that nifuroxazide inhibited breast cancer cells viability in a concentration- and time-dependent manner.

To further investigate whether nifuroxazide could inhibit viability of breast cancer cells, we conducted clonogenic assay after nifuroxazide treatment. As shown in Figure 1b and Supplementary Figure S1b, clonogenic assay clearly showed that clone formation of 4T1, MCF-7 and MDA-MB-231 cells was reduced in a concentration-dependent manner after exposure to nifuroxazide. Moreover, the size of the colonies treated with nifuroxazide was significantly smaller than the control. These results were consistent with the MTT data. Taken together, those results suggested that nifuroxazide had a strong cytostatic and cytotoxic effects on breast cancer cells.

### Breast cancer cells apoptosis induced by nifuroxazide

**Figure 2:**
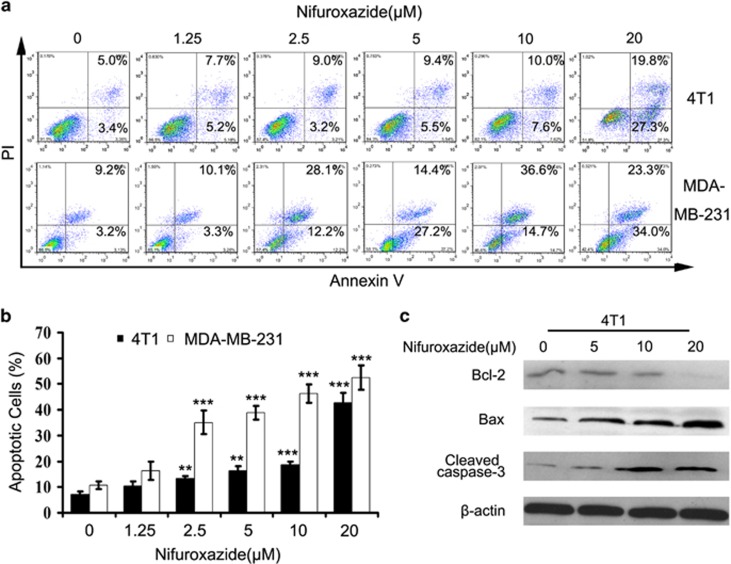
Nifuroxazide induces breast cancer cells apoptosis. (**a**) 4T1 and MDA-MB-231 cells were treated with nifuroxazide at indicated doses for 24 h, and the level of apoptosis was evaluated using the Annexin V/PI dual-labeling technique, as determined by FCM. Data shown are representative of three independent experiments. (**b**) Statistic results of apoptosis assays, LR represents early apoptotic cells (positive for Annexin V only) and LL represents live cells. Graphical presentation of data obtained by Annexin V/PI staining after nifuroxazide treatment was also shown. Data are expressed as mean±S.D. from three independent experiments (*P<0.05; **P<0.01; ***P<0.001). (**c**) Western blot analyses of 4T1 cells treated (24 h) with different concentrations of nifuroxazide to evaluate protein expression of Bcl-2, cleaved caspase-3 and *β*-actin was used as a standard

We next explored whether nifuroxazide induced breast cancer cells apoptosis. Hoechst 33258 staining assay showed that nifuroxazide treated induced apoptosis in 4T1, MCF-7 and MDA-MB-231 cells, with the features of a bright-blue fluorescent-condensed nuclei and nuclear fragmentation (Supplementary Figure S1c). To further confirm the induction of apoptosis in 4T1 and MDA-MB-231 cells with nifuroxazide treatment, we also investigated the levels of apoptosis by low cytometry (FCM) using the Annexin V-FITC/PI dual-labeling technique. As shown in Figures 2a and b, after nifuroxazide treatment for 24 h, the apoptosis induction effects were observed. When the 4T1 cells were treated with 1.25 *μ*M nifuroxazide, the apoptosis rate was 7.0%, whereas the apoptosis cells increased to 10.3, 13.4, 16.2, 18.7 and 42.8% when cells were treated with 1.25, 2.5, 5, 10 and 20 *μ*M nifuroxazide, respectively, indicating that nifuroxazide was able to induce apoptosis in a concentration-dependent manner. Similarly, in MDA-MB-231 cells, the percentage of apoptotic cells was increased from 10.7 to 16.3, 35.1, 38.9, 46.3 and 52.6% after treatment with various concentrations of nifuroxazide for 24 h.

To further confirm the characterization of nifuroxazide-induced apoptosis, some apoptosis-related proteins were detected by western blot. We examined Bcl-2, Bax and cleaved caspase-3 (CC-3) expression levels in 4T1 cells after nifuroxazide treated for 24 h. As shown in Figure 2c, the expression of Bcl-2 significantly decreased, whereas that of CC-3 and Bax increased in a concentration-dependent manner and a significant increase in the ration of Bax/Bcl-2 (Supplementary Figure S2), suggesting that nifuroxazide-induced apoptosis might be via the mitochondrial apoptotic pathway. Collectively, those results showed that nifuroxazide could induce the apoptosis of breast cancer cells.

### Nifuroxazide inhibits breast cancer cell migration and invasion

**Figure 3:**
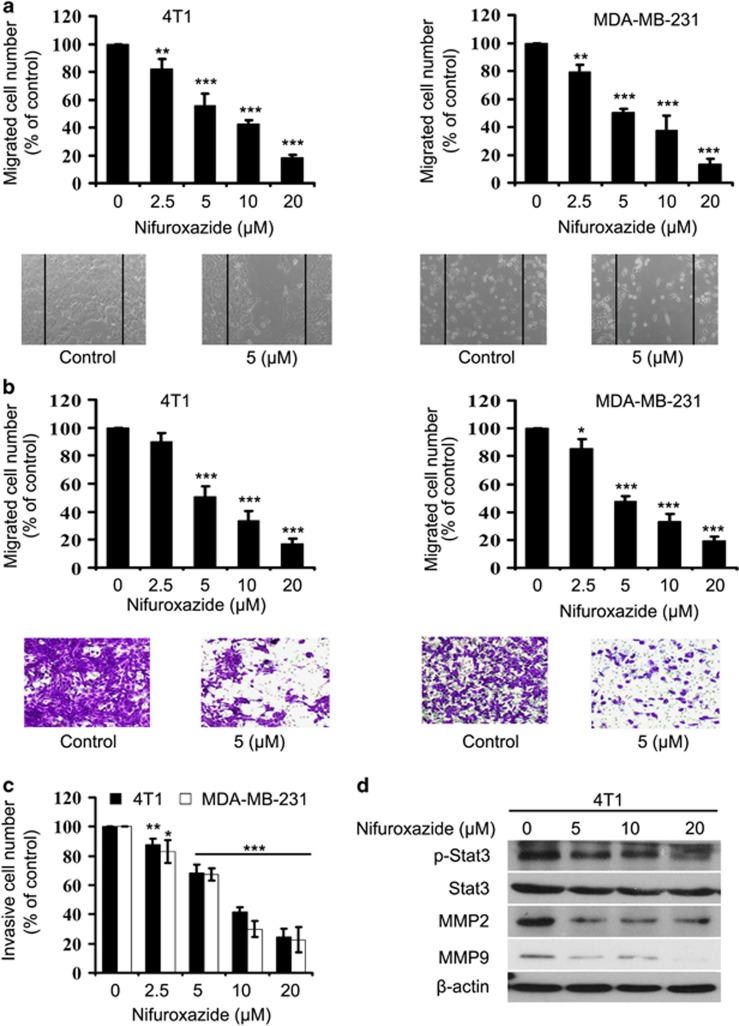
Nifuroxazide inhibits breast cancer cells 4T1 and MDA-MB231 migration and invasion. (**a**) Nifuroxazide inhibits 4T1 and MDA-MB231 migration. Tumor cells were seeded in six-well plates. A ‘wound' was created after the cells grew ~85% confluence. After incubation for 24 h the groups were fixed and photographed. The lines indicate the area occupied by the initial scraping, and migrated cells were quantified. (**b**) Tumor cells were seeded in the top chamber of transwell with serum-free medium and treated with vehicle or various concentrations of nifuroxazide. After about 24 h, migrated cells were fixed, stained and photographed (10 × ) and quantified. (**c**) Nifuroxazide inhibits 4T1 and MDA-MB231 invasion. Tumor cells were treated with different concentrations of nifuroxazide and allowed to invade through Matrigel. Invaded cell number was counted (*P<0.05; **P<0.01; ***P<0.001). (**d**) 4T1 cells were treated with different concentrations of nifuroxazide. After 24 h, cells were harvested, and western blot assay was performed to test the expression of MMP-2, MMP-9 and P-Stat3. *β*-actin served as loading control

One of the key steps in successful breast cancer metastasis is cancer cell migration and invasion.^28, 29^ Therefore, it is imperative to investigate whether nifuroxazide could inhibit breast cancer cell migration and invasion. To assess the effects of nifuroxazide on migration, we performed wound-healing and transwell migration assays using 4T1 and MDA-MB-231 cell lines. As shown in Figure 3a, nifuroxazide inhibits migration of both 4T1 and MDA-MB-231 cells in dose-dependent manners. Similar results were obtained in transwell migration assay (Figure 3b).

We then performed a Matrigel invasion assay. Figure 3c shows that both 4T1 and MDA-MB-231 cells exhibit significantly decreased invasion in the presence of nifuroxazide than vehicle. Moreover, constitutive Stat3 also has an important role in controlling cell migration and invasion by regulating the expression of genes involved MMP-2, -9 and others.^30^ Therefore, we also investigated whether phosphorylated-Stat3 ^(Tyr705)^, MMP-2, -9, which are considered to be related with cell migration and invasion, are involved in nifuroxazide-mediated migration and invasion. As Figure 3d indicates, nifuroxazide treatment decreased the expression of phosphorylated-Stat3 ^(Tyr705)^, MMP-2 and MMP-9 without affecting the total Stat3 expression level in 4T1 cells. Altogether, our results showed that nifuroxazide possessed a strong ability on breast cancer cell migration and invasion.

### Retardation of mammary tumor growth *in vivo*

**Figure 4:**
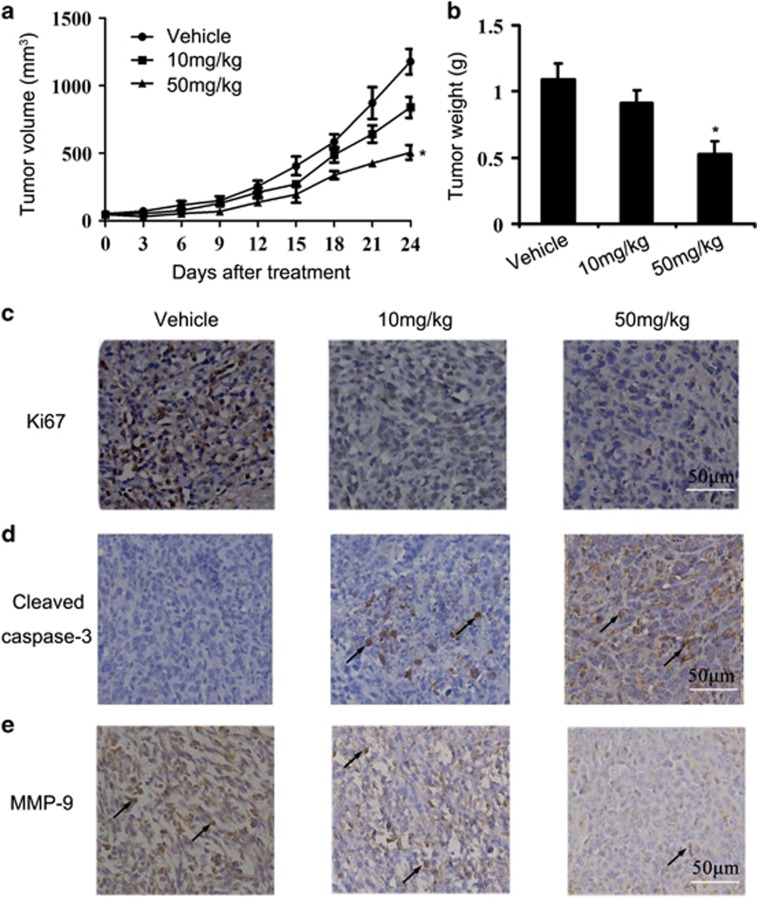
Antitumor effects of nifuroxazide *in vivo*. (**a**) Tumor size were measured and calculated every 3 days and presented as mean±S.D. (*n*=6; *P<0.05). (**b**) Represented weight of tumor from mice of different groups, respectively. Data were mean ±S.D. (*n*=6; *P<0.05). (**c**) Tumor cell proliferation was evaluated on paraffin-embedded 4T1 tumor sections by Ki-67 immunohistochemical staining. The treatment with nifuroxazide resulted in markedly reduced proliferation *versus* vehicle group. (**d**) Apoptosis was measured on paraffin-embedded 4T1 tumor sections by CC-3 immunohistochemical staining. The treatment with nifuroxazide significantly increased apoptosis in a dose-dependent manner compared with vehicle group. (**e**) Immunohistochemistry was performed to measure the expression of MMP-9 in tumor tissues isolated from vehicle and nifuroxazide-treated mice. The treatment with nifuroxazide markedly reduced MMP-9-positive cells *versus* vehicle group (20 × )

The remarkable inhibitory effects of nifuroxazide on 4T1 cells proliferation *in vitro* implied that it might inhibit tumor growth *in vivo*. To verify this hypothesis, we examine the antitumor activity of nifuroxazide *in vivo*. 4T1 tumor-bearing mice were dosed daily at the dose of 10 and 50 mg/kg for 24 days. Nonsignificant changes in body weight were observed in nifuroxazide-treated animals (Supplementary Figure S3a). Importantly, there was a significant reduction in tumor outgrowth and in tumor weight in the nifuroxazide-treated groups (Figures 4a and b; Supplementary Figure S3b), showing that nifuroxazide has strong antitumor activity.

To determine the mechanisms underlying nifuroxazide activity, 4T1-induced tumors collected on day 31 were examined for proliferation and apoptosis. Nifuroxazide treatment caused a significant reduction in proliferating cells stained positive for nuclear Ki-67 (Figure 4c). Moreover, as shown in Figure 4d, CC-3-positive cells were showing an increase in the nifuroxazide-treated sections *versus* sections of the untreated groups. Overall, these data suggest that nifuroxazide inhibits cell proliferation and induces apoptosis in breast tumor tissues through inhibition of Ki-67 and activation of caspase-3. In addition, there is increasing evidence to indicate that MMPs have important roles in tumor invasion and metastasis.^31^ We therefore measured the effects of nifuroxazide on the expression of MMP-9 by immunohistochemistry (IHC). As shown in Figure 4e, treatment of mice with nifuroxazide inhibited the expression of MMP-9 in 4T1 tumor tissues.

### Nifuroxazide treatment decreases lung metastasis

**Figure 5:**
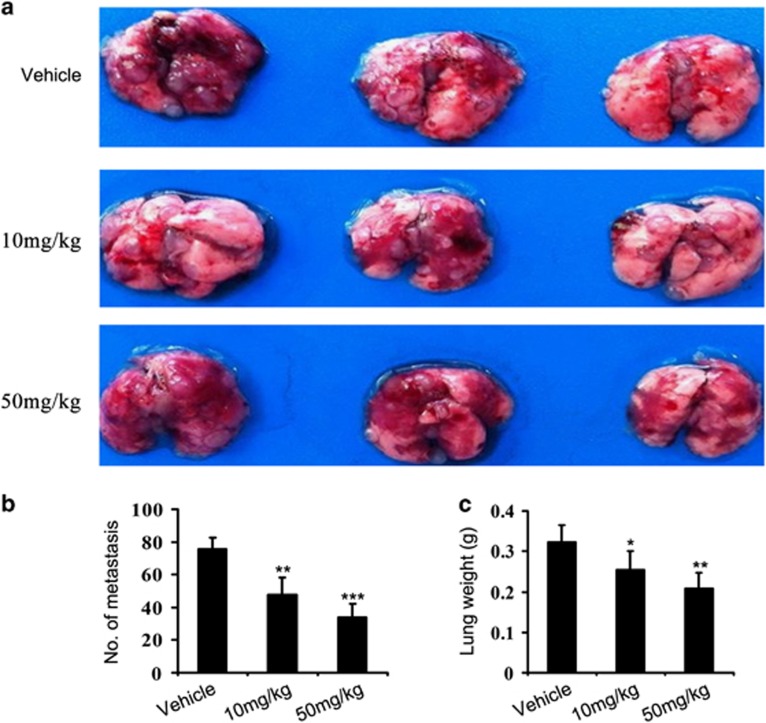
Effects of nifuroxazide on metastasis. (**a**) Lung metastatic nodules were visualized to show the inhibitory effects of nifuroxazide on 4T1 tumor 24 days after treatment. (**b**) The mean lung metastasis nodules of each group, the treatment with nifuroxazide at 10 mg/kg and 50 mg/kg resulted in significant inhibition of lung metastasis *versus* vehicle control. Bars showed mean±S.D. (*n*=6; **P<0.01; ***P<0.001). (c) Weight of lungs in each group. Bars showed mean±S.D. (*n*=6; *P<0.05; **P<0.01)

Previous studies have showed that 4T1 murine breast tumor have a high metastatic potential and spontaneously metastasize to lung as early as 2 weeks after inoculation.^32, 33, 34^ To analyze the effects of nifuroxazide on metastasis, lungs of 4T1 tumor-bearing mice killed on day 31 were removed and metastatic nodules were quantified. In vehicle-treated groups, multiple large nodules were evident, whereas the extent of lung metastasis was markedly reduced in nifuroxazide-treated mice (50 mg/kg groups; Figures 5a and b). Moreover, there was a remarkable decrease in lung weight after nifuroxazide treatment compared with the untreated control (Figure 5c). Importantly, histological analyses demonstrated that the number of micrometastatic nodules per field in the nifuroxazide-treated group at 50 mg/kg was also significant fewer than the other groups (Supplementary Figure S4). Overall, these results further indicated that nifuroxazide could inhibit lung metastasis in breast cancer.

### Nifuroxazide modulates the lung metastatic environment

**Figure 6:**
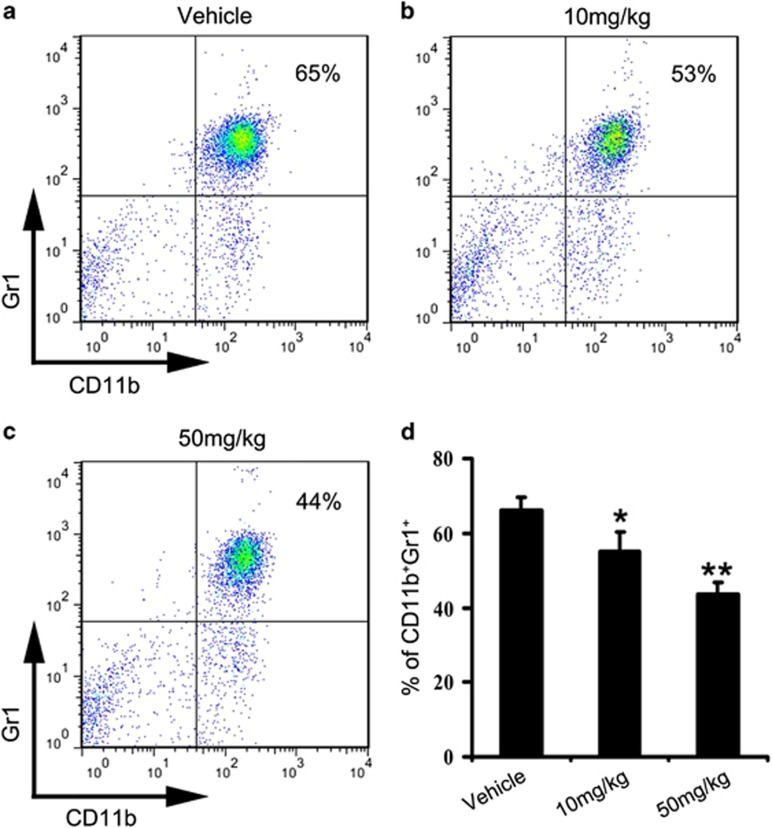
Nifuroxazide reduced lung Gr1^+^CD11b^+^MDSCs infiltration. Gr1^+^CD11b^+^cells were gated and analyzed by FCM for the expression of MDSCs. MDSCs isolated from the lungs of 4T1 tumor-bearing mice were treated with vehicle (**a**); or treated with nifuroxazide at 10 mg/kg (**b**); or treated with nifuroxazide at 50 mg/kg (**c**). (**d**) Statistic results of each group. Treatment of nifuroxazide significantly reduces the number of MDSCs compared with vehicle group. Values represented mean±S.D. (*n*=6; *P<0.05; **P<0.01)

MDSCs, as mainly characterized by CD11b^+^ and Gr1^+^ double-positive myeloid cells in mice have been observed to accumulate in highly metastatic breast carcinoma 4T1. Moreover, it has been shown that accumulation of MDSCs into the lung has a key role in the development of metastasis, meanwhile, MDSCs are closely related to a lung metastatic in patients with breast cancer.^35, 36, 37^ Therefore, we further investigated lung myeloid cells infiltration in 4T1 tumor-bearing mice by FCM after 24 days of treatment. As shown in Figures 6a and b, the data showed that the percentage of MDSCs decreased in the 10 mg/kg-treated group compared with the control group. Moreover, we found that ~22% reduction of MDSCs in the lung after 50 mg/kg nifuroxazide treatment (Figure 6c). The statistical analysis demonstrated that nifuroxazide treatment reduced the number of MDSCs in lung in a dose-dependent manner (Figure 6d). These results suggested that nifuroxazide potently reduced the infiltration of MDSCs into the lung, which could inhibit tumor cell distant colonization.

### Safety profile of nifuroxazide

**Figure 7:**
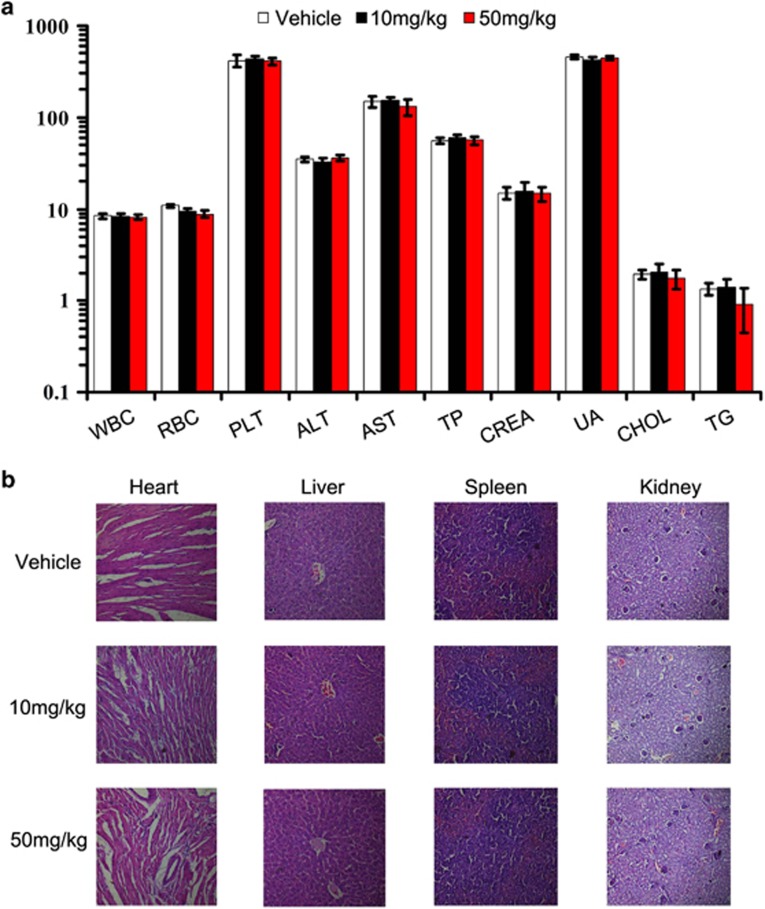
Evaluation of side effects of nifuroxazide in mice. (**a**) Hematological and serum biochemistry analysis of blood were done. Units of the parameters are as follows. WBC, PLT, 10^9^/l; RBC, 10^12^/l; TP, g/l; ALT, AST, U/l; CREA, UA, uM; TG, CHOL, mM. (**b**) Nifuroxazide did not cause obvious pathologic abnormalities in normal tissues. H&E staining of paraffin-embedded sections of the heart, liver, spleen and kidney (20 × )

As mentioned above, during the treatment of 4T1 tumor-bearing mice, we did not observed adverse effects, such as toxic death, skin ulceration and body weight loss, in the nifuroxazide-treated groups. To further investigate the safety profile of nifuroxazide, we determined here whether the nifuroxazide could cause the blood system's abnormality, we performed blood routine analysis and blood chemistry analysis assays. Hematological and serum biochemistry analysis of the mice did not show any pathological changes (Figure 7a). Furthermore, no pathologic changes after nifuroxazide treatment were observed in the heart, liver, spleen and kidney by microscopic examination compared with the vehicle-treatment group (Figure 7b).

## Discussion

Breast cancer is highly malignant with considerable metastatic potential. Meanwhile, tumor metastasis poses a predominant threat to cancer-related mortality.^29^ Despite breast cancer is the most frequently diagnosed cancer and recent advances in the treatment of breast cancer, there are patients for whom no targeted therapies are available.^38^ Therefore, the discovery of novel potential drug candidate to prevent tumor metastasis is still needed. The association between breast cancer and Stat3 pathway was established about 20 years ago.^39^ Recent studies have also reported that activated Stat3 and its overexpression were closely associated with the development of breast cancer.^40^ Importantly, with Stat3 being activated in ~70% of breast tumors, particularly in the less treatable triple-negative tumors, suggesting that targeting Stat3 might be a potentially important new form of breast cancer therapy.^14^

In the present study, we reported our finding with nifuroxazide, an antidiarrheal agent. Our results showed that nifuroxazide could inhibit breast cancer cell vitality with low micromole. Proliferation inhibitory activity of nifuroxazide against cancer cells was confirmed by MTT and clonogenicity assays. Apoptosis has been accepted as a fundamental component in the pathogenesis of cancer, and it has an important role in breast cancer progression. Therefore, inducing apoptosis is a therapeutic approach to treat cancers.^41, 42^ Our data indicated that nifuroxazide induced apoptotic death in breast cancer cells in a concentration-dependent manner, which was confirmed by the downregulation of Bcl-2 and the upregulation of CC-3 and Bax.

Because not all compounds have potent antitumor activity *in vitro* could exhibit anticancer activity *in vivo*, we then examined the antitumor effects of nifuroxazide in our established 4T1 tumor model in BALB/c mice. The results showed that tumor growth was significantly inhibited by nifuroxazide administration (50 mg/kg/day) with an inhibitory rate by 65%. Meanwhile, reduced expression of Ki-67 and increased expression of CC-3 in tumor cells were observed after nifuroxazide treatment compared with the untreated groups.

Breast cancer starts as a local disease, but it can metastasize to the lymph node, lung and other organs.^29^ The metastatic process is complex, and tumor cells need to enter into blood vessels as well as the extravasation into the secondary organs. Therefore, tumor cell migration and invasion is a key step in successful cancer metastasis, and inhibition of this step is a practical approach to antitumor treatment.^43^ Nifuroxazide was shown to inhibit MDA-MB-231 and 4T1 cell migration and invasion. In addition, in our animal experiments, administrations of nifuroxazide at the dose of 50 mg/kg significantly inhibited breast cancer metastasis to lung. These results consist with *in vitro* experiment, suggesting that breast cancer metastasis inhibition by nifuroxazide could be mainly ascribed to the impediments of tumor cell migration and invasion. Moreover, it has been reported that focal adhesion kinase/MMP-involved pathway is critical for cancer invasion and metastasis.^28, 44^ MMP-2 and MMP-9 upregulation can particularly enhance tumor cell metastatic potential in breast cancer.^45^ In addition, constitutive Stat3 also has an important role in controlling cell migration and invasion.^30^ In this study, nifuroxazide not only decreased expression of Stat3 phosphorylation at tyrsione residue 705 but also downregulated MMP-2 and MMP-9 expression in 4T1 cells. Meanwhile, IHC assay results showed that nifuroxazide inhibited the expression of MMP-9 *in vivo*. The results suggested the efficacy of MMPs signaling pathway blockade by nifuroxazide for inhibition of breast cancer cell invasion and metastasis.

A large body of evidence suggests that the tumor microenvironment can promote tumor development, progression and immune evasion.^46, 47^ MDSCs are critical components of the tumor microenvironment. In addition, MDSCs are present in lots of patients and experimental animals with cancer that downregulate immune surveillance and antitumor immunity.^48^ In this study, our results indicated that the treatment of mice with nifuroxazide caused a significant decrease in the number of MDSCs in lung compared with that of vehicle-treated group. It is therefore conceivable that nifuroxazide could potentiate the antitumor effects and suppress the lung metastasis by downregulating the number of MDSCs.

In summary, our present studies provide important information regarding the antitumor activities of nifuroxazide in breast cancer. To our knowledge, this is the first study, to demonstrate that nifuroxazide could inhibit breast cancer cell growth, induce cell apoptosis and block cell migration and invasion. Moreover, nifuroxazide suppressed the breast tumor growth without significant toxicity. Importantly, nifuroxazide could enhance antitumor immunity and inhibit lung metastasis by reducing the number of MDSCs in lung. Therefore, these results implied that nifuroxazide might be a potential therapeutic agent for blocking breast cancer growth and metastasis.

## Materials and Methods

### Reagents and antibodies

Nifuroxazide was purchased from (Xiyashiji Chemical Co., LTD, ChengDu, Sichuan, China). Purity (98%) was measured by high-performance liquid chromatograph analysis. For *in vitro* assays, nifuroxazide was prepared initially as a 20 mM stock solution in dimethyl sulfoxide (DMSO) and stored at −20 °C. Then the stock solution diluted in the relevant assay medium, and 0.1% DMSO served as a vehicle control. For *in vivo* studies, nifuroxazide was prepared in 40% (v/v) polyethylene glycol 400 containing 5% (v/v) propylene glycol and dosed at 0.1 ml/10 g of body weight.

MTT, DMSO and Hoechst 33258 were from Sigma Chemical Co. (St Louis, MO, USA). The primary antibodies against Stat3/P-Stat3^Tyr705^, MMP-2, -9 (MMP-2, MMP-9), CC-3, Bcl-2 and *β*-actin were purchased from Cell Signaling Technology (Beverly, MA, USA). FITC-CD11b, PE-Gr1 conjugated antibodies were obtained from BD Biosciences (San Diego, CA, USA). Mouse monoclonal anti Ki-67 was purchased from Merck-Millipore (Bill, MA, USA). The Annexin V-FITC Apoptosis Detection Kit was purchased from KeyGen Biotech (Nan-jing, China).

### Cell culture

The human breast cancer cell lines, MDA-MB-231 and MCF-7, the mouse mammary carcinoma cell line 4T1, were obtained from the American Type Culture Collection (Rockville, MD, USA). Cells were propagated in DMEM or RPMI 1640 media containing 10% heat-inactivated fetal bovine serum (FBS; Hyclone, Logan, UT, USA) and 1% antibiotics (penicillin and streptomycin) in 5% CO_2_ at 37 °C.

### Cell viability assay

The cell viability of nifuroxazide-treated breast cancer cells was assessed by MTT assay. In brief, the exponentially growing cells (3~5 × 10^3^/well) were plated in 100 *μ*l/well in 96-well plates. After 24 h incubation, the cells were treated with different concentrations of nifuroxazide. After treatment for 24, 48 and 72 h, respectively, the 20 *μ*l of 5 mg/ml MTT was added to each well, and the plates were incubated at 37 °C for additional 2–4 h. The medium was subsequently removed, the purple-colored precipitates of formazan were dissolved in 150 *μ*l of DMSO. The color absorbance was recorded at 570 nm using a Spectra MAX M5 microplate spectrophotometer (Molecular Devices, CA, USA). All experiments were performed in triplicate.

### Colony formation assay

Colony formation assay was measured as previously described.^49^ In brief, 4T1, MCF-7 and MDA-MB-231 cells were seeded in specified numbers (400–600 cells/well) in six-well plates. After 24 h incubation, the cells were treated with various concentrations of nifuroxazide and then incubated for additional 12 days. Then the cells were fixed with methanol and stained with a 0.5% crystal violet solution for 15 min, and the colonies (>50 cells) were counted under microscope. Data shown represent the average of three independent experiments.

### Morphological analysis by Hoechst staining

An apoptotic cell has special morphologic characteristics such as cell body shrinkage, chromatin condensation and margination as well as emerging apoptotic bodies.^49^ To identify whether the nifuroxazide-inducing reduction in cell viability was attributable to the apoptosis, we stained the 4T1, MCF-7 and MDA-MB-231 cells with Hoechst 33258 dye. In brief, 4T1, MCF-7 and MDA-MB-231 cells (1~2 × 10^5^cells/well) were plated onto 18-mm coverglass in a six-well plate for 24 h, respectively. After treatment with different concentrations for following 24 h, the cells were washed with cold phosphate-buffered saline (PBS) and fixed in methanol for 15 min. The cells were stained with the Hoechst 33258 solutions according to the manufacturer's instructions. Then nuclear morphology of apoptotic cells was examined under a fluorescence microscopy (Leica, DM4000B, Wetzlar, Germany).

### Apoptotic assay

To further confirm the apoptosis-inducing effects of nifuroxazide, we subsequently estimated the number of apoptotic cells by FCM. In brief, breast cancer cells (1~2 × 10^5^ cells/well) were seeded in six-well plate overnight. Then treated with various concentrations of nifuroxazide for 24 h, the cells were harvested, washed with cold PBS twice. The apoptosis levels were examined using an apoptosis detection kit according to manufacturer's instructions by FCM. Then data were analyzed with FlowJo software.

### Flow cytometry

Single-cell suspensions from lung were prepared as described previously.^15^ Then 1 × 10^6^ freshly prepared cells were stained with fluorochrome-conjugated CD11b and Gr1 antibodies. Data were collected by FCM and analyzed with FlowJo software.

### Western blot analysis

The western blot analysis was performed as described previously, with minor modification.^50^ in brief, 4T1 cells were treated with nifuroxazide in designed concentration for 24 h, then cells were washed twice with cold PBS and lysed in RIPA buffer. The protein concentrations were measured using the Lowry method and equalized before loading. Equal amounts of protein from each sample were subjected to sodium dodecyl sulfate-polyacrylamide gel electrophoresi gels and transferred onto polyvinylidene difluoridemembranes (Amersham Bioscience, Piscataway, NJ, USA). Then, the membranes were blocked for 2 h at 37 °C and incubated with specific primary antibodies overnight at 4 °C. After incubation with the relevant secondary antibodies, the reactive bands were identified using an enhanced chemiluminescence kit (Amersham).

### Wound-healing migration assay

Wound-healing migration assay was performed as described previously.^28^ When cancer cells grew to 80% confluence, cell monolayer was scraped by sterile 0.1 ml pipette tips, and fresh medium was added containing different concentrations of nifuroxazide. After 24 h incubation, cells were fixed and photographed. Images were acquired using a microscope (Zeiss, Jena, Germany) and the percentage inhibition of migrated cells was expressed using 100% as the value assigned for untreated group.

### Boyden chamber migration and invasion assay

Boyden chamber (8 *μ*m pore size) migration assay was performed as previously described, with some modification.^22, 28^ In brief, a total of 1 × 10^5^ cells (for 4T1) or 5 × 10^4^ cells (for MDA-MB-231) in 100 *μ*l serum-free medium were added in the upper chamber, and 600 *μ*l of medium containing 10% FBS was added at the bottom. Different concentrations of nifuroxazide were added in both chambers. Cells were allowed to migrate for ~24 h. Non-migrated cells in the upper chamber were discarded using a cotton swab. The migrated cells were fixed in methanol and stained with 0.5% crystal violet. Migrated cells in six randomly selected fields were counted and photographed under a light microscope. Invasion assay was performed according to previous studies.^22, 28^ In brief, the upper surface of the transwell membrane were coated with serum-free medium diluted Matrigel (1 : 1, 60 *μ*l/well, BD Biosciences) and the lower compartment of the chambers were filled with 600 *μ*l medium with 10 % FBS. 1 × 10^5^ cells (for 4T1) or 5 × 10^4^ cells (for MDA-MB-231) in 100 *μ*l serum-free medium were placed in the upper part of each transwell and treated with different concentrations of nifuroxazide. After incubation for 24 h, cells on the upper side of the filter were removed. Cells located on the underside of the filter were fixed with methanol and stained with 0.5% crystal violet, then, migrated cells were counted and photographed under a light microscope. The results were expressed as the percentage inhibition rate of migration compared with untreated group.

### Mice and tumor model

All animal experiments were approved by the Institutional Animal Care and Treatment Committee of Sichuan University in China (New Permit Number: 20131205-3). Female BALB/c mice (6–8 weeks old) were obtained from HFK bioscience CO., LTD, Beijing, China. We inoculated female mice with 4T1 cell (1.0 × 10^6^/100 *μ*l/each). After ~4 days, the injection sites could appear tumors. When visible tumors (~70 mm^3^) had been developed at the injection sites, the tumor-bearing mice were randomized into three groups (eight mice per group), and received intraperitoneally injection of nifuroxazide 50 mg/kg, 10 mg/kg or vehicle, respectively, once daily for 24 days. Tumor volumes and body weight were measured every 3 days. The tumor size was calculated according to the formula: Tumor volume (mm^3^)=0.52 × *L* × *W*^2^ where *L* is the length and *W* is the width. At the termination of the experiment, all animals were euthanized by cervical dislocation. At that time tumors and internal organs, such as the heart, livers, spleens, lungs and kidney were excised from animals.

### Immunohistochemistry

IHC staining of tumor sections were described previously.^22, 28^ One part of paraffin tumor sections and lung sections were stained with hematoxylin and eosin (H&E). The other part paraffin tumor sections were stained with Ki-67, CC-3 and MMP-9 antibodies. Images were taken with Leica microscope (Leica, DM4000B).

### Toxicity evaluation

To investigate potential side effects or toxicity on mice during the treatment, all the animals were observed continuously for relevant indexes such as body weight, diarrhea, anorexia and other clinical symptoms. On the 31st day, all animals were euthanized by cervical dislocation after taking blood from eyeball. Blood was obtained for blood routine analysis and blood chemistry analysis. The tissues of heart, liver, spleen, lung and kidney were stained with H&E for histopathologic examination.

### Statistical analysis

Data were represented as mean±S.D. of three independent experiments. The two-tailed Student's *t-*test was used for statistical analysis and statistically significant *P-*values were labeled as follows: *P<0.05; **P<0.01; ***P<0.001.

## Abbreviations

Stat3: Signal transducers and activators of transcription 3
MTT: 3-(4, 5-dimethylthiazol-2-yl)-2, 5-diphenyltetrazolium bromide
FCM: flow cytometry
MMP: matrix metalloproteinase
IHC: immunohistochemical
MDCSs: myeloid-derived suppressor cells
TNBC: triple-negative breast cancer
ER: estrogen receptor
PR: progesterone receptor
HER2: human epidermal growth factor receptor 2
VEGF: vascular endothelial growth factor
HPLC: high-performance liquid chromatography
DMSO: dimethyl sulfoxide
CC-3: cleaved caspase-3
FBS: fetal bovine serum
PBS: phosphate-buffered saline
SDS-PAGE: sodium dodecyl sulfate-polyacrylamide gel electrophoresis
PVDF: polyvinylidene difluoride
H&E: hematoxylin and eosin
FAK: focal adhesion kinase
i.p.: Intraperitoneally
