# Supplementary material for: Nifuroxazide induces apoptosis and impairs pulmonary metastasis in breast cancer model
Source: Cell Death Dis. 2015 Mar 26;6(3):e1701–. doi: 10.1038/cddis.2015.63 (PMC4385941; doi:10.1038/cddis.2015.63)

**Supplementary Figure 1. The effects of nifuroxazide on breast cancer cells viability.** (a) Equal protein amounts from breast cancer cell lines were analyzed by western blot for phosphorylated Stat3 (p-Stat3) expression levels. (b) The effects of nifuroxazide (0-1 μM) on colony formation in MCF-7 cells for 12 days. (c) The fluorescence microscopic appearance of Hoechst 33258 staining nuclei of 4T1, MDA-MB-231 and MCF-7 cells with various concentration nifuroxazide for 24 h.


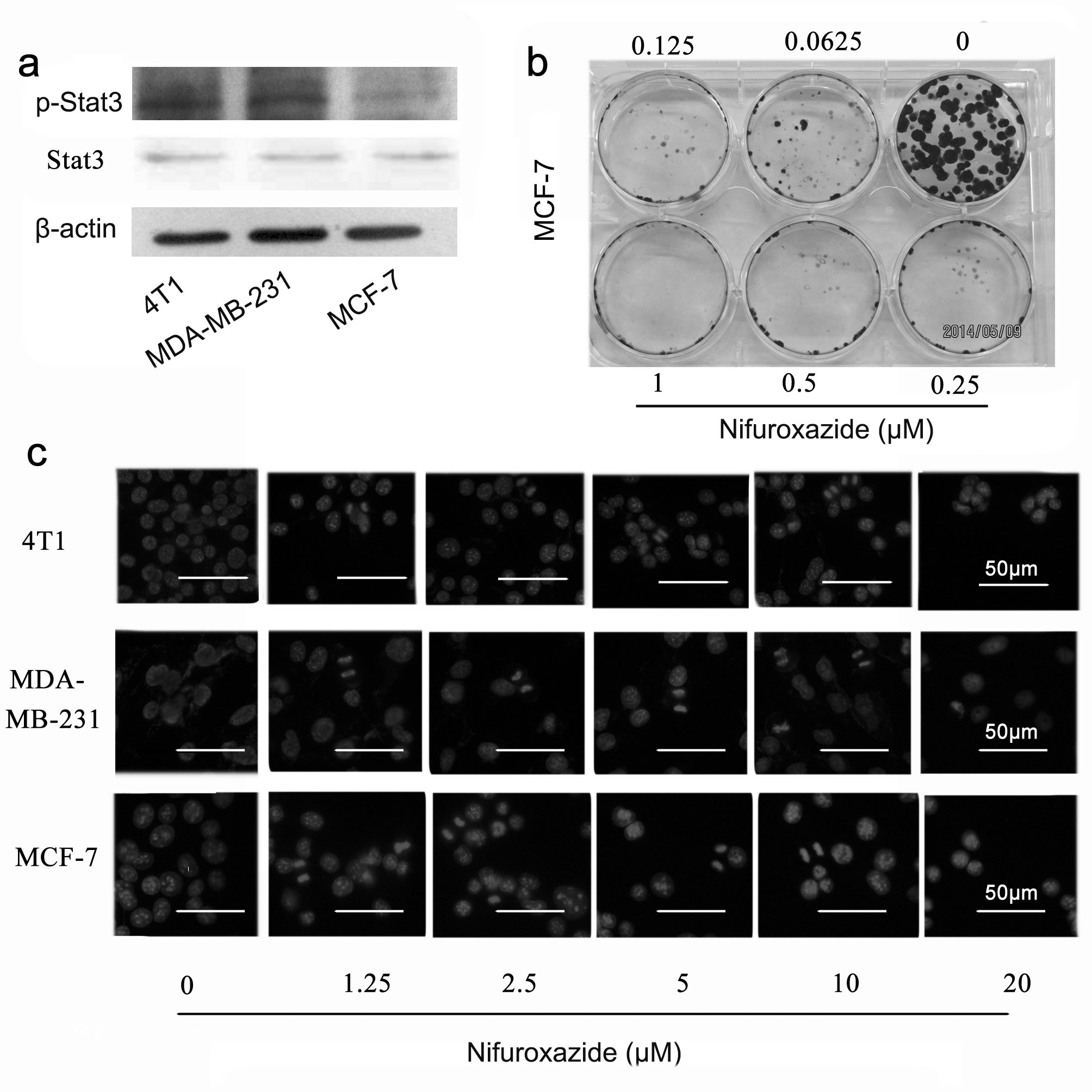


**Supplementary Fig. 2** **Percentage of Bax/Bcl-2 ratio.** The data were presented in the bar graphs as percentage of Bax/Bcl-2 ratio. Columns, means of three experiment; bars, SD. **P* < 0.05 ,***P* < 0.01 and ****P* < 0.001 versus the control.


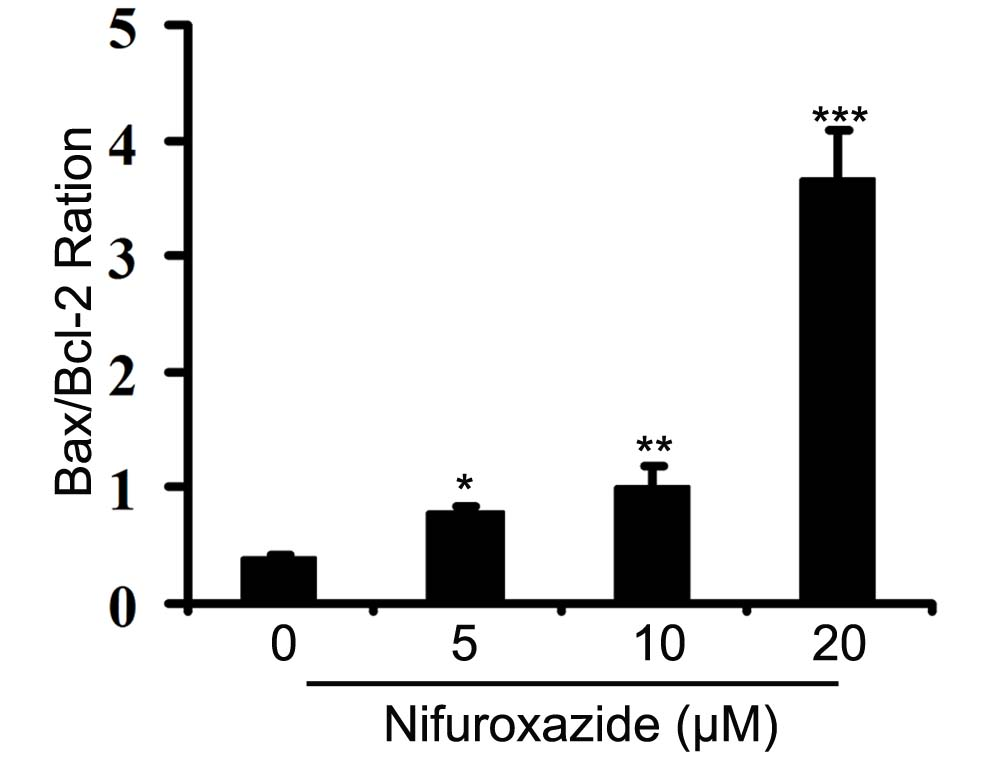


**Supplementary Fig. 3 Nifuroxazide inhibited tumor growth in subcutaneous 4T1 model.** (a) 24 days after nifuroxazide treatment, body weight had no signiﬁcant difference. (b) Representative photographs of subcutaneous tumors in each group.


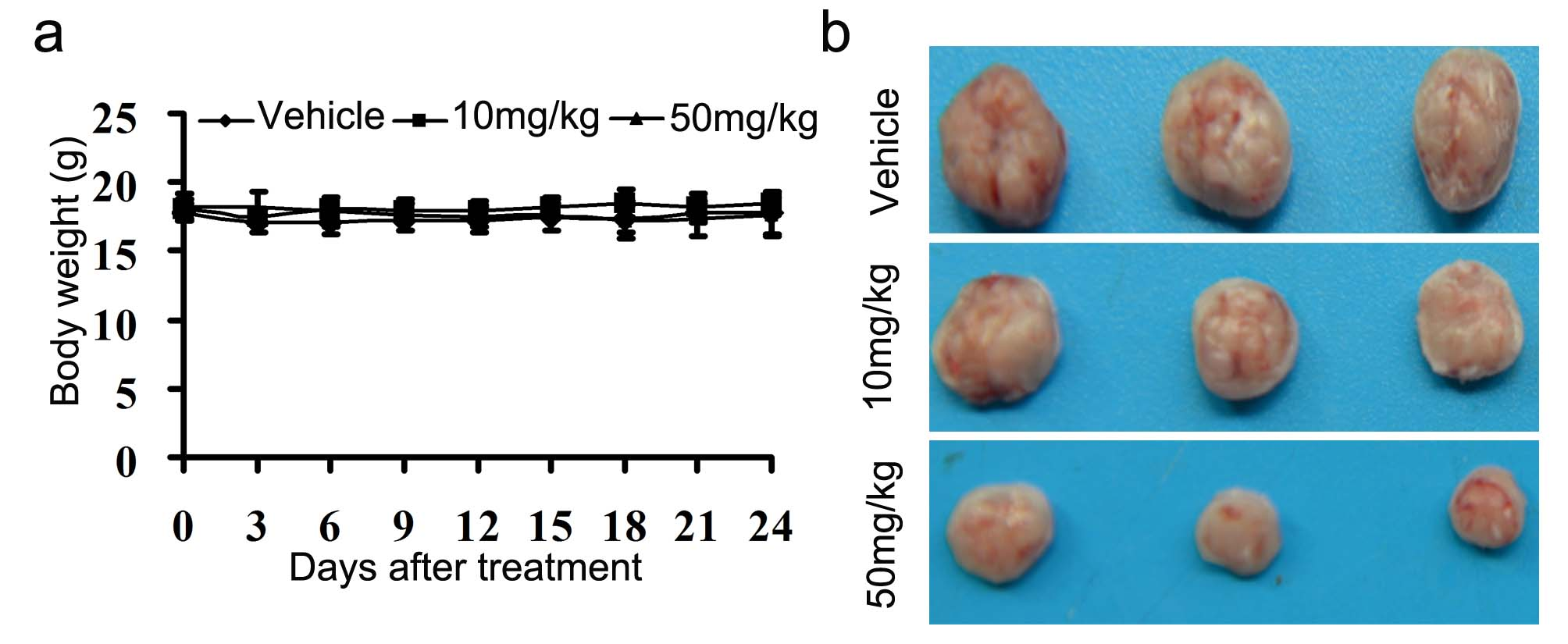


**Supplementary Fig. 4 Nifuroxazide inhibited spontaneous lung metastasis of mouse syngeneic tumors.** H&E staining of lung tissues harvested from 4T1 tumor-bearing mice and treated with nifuroxazide or vehicle for 24 days (10×).


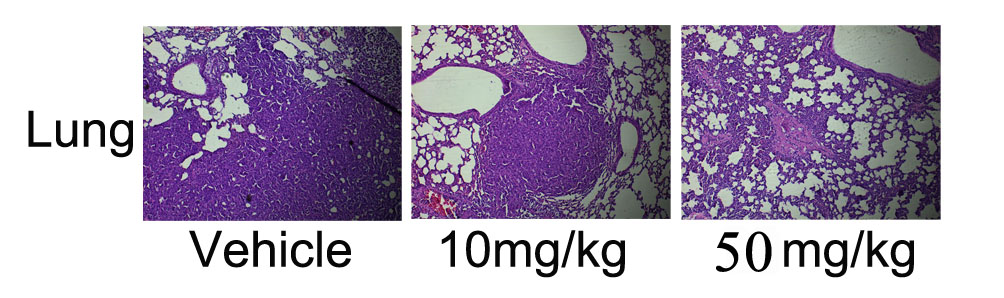

Supplement: Supplementary Figures [file cddis201563x1.doc]
